# Rapid cardiac T1 mapping within two heartbeats

**DOI:** 10.1186/1532-429X-13-S1-O107

**Published:** 2011-02-02

**Authors:** Elodie Breton, Daniel Kim, Sohae Chung, Leon Axel

**Affiliations:** 1 NYU Langone Medical Center, New York, NY, USA

## Introduction

Late gadolinium enhancement (LGE) imaging is an important CMR method than can detect salvageable myocardium after myocardial infarction [1-2]. Recently, T_2_-weighted-imaging has gained a significant interest to assess myocardial edema [3]. However, clinical interpretation of T_2_-weighted-imaging could be hindered by surface coil effects which yield non-uniform signals. Multi-point T_1_ mapping approaches, such as Modified Look-Locker inversion recovery (MOLLI) [4], have been proposed to measure myocardial T_1_, but, as a multiple heartbeat acquisition, it may be sensitive to cardiac motion and arrhythmia. We propose to develop a 2-second cardiac T_1_ mapping pulse sequence for assessment of myocardial edema (pre-contrast) and infarction (post contrast) in patients with acute myocardial infarction.

## Purpose

To develop and validate a cardiac T_1_-mapping technique.

## Methods

The proposed T_1_-mapping acquisition consists of 2 TurboFLASH images with centric k-space ordering: proton density-weighted (PDw) image in the first heartbeat and saturation recovery (SR) T_1_w acquisition in the second heartbeat. A robust non-selective saturation pulse [5] was used to achieve uniform saturation of magnetization. A long delay time=500ms was used to achieve adequate signal-to-noise ratio. The T_1_w-image was normalized by the PDw image to correct for unknown equilibrium magnetization and receiver coil sensitivity. T_1_ was calculated algebraically assuming an ideal saturation-recovery equation based on the Bloch equation [6]. Eight healthy volunteers (32±13y.o.) were imaged in a short-axis basal plane at 3T (Tim-Trio, Siemens) at baseline and 10 minutes following 0.05mmol/kg Gd-DTPA injection. All images were acquired in mid-diastole with appropriate trigger delay. Imaging parameters included: FOV=350mm×272mm, matrix=144×112, TE/TR=1.2/2.4ms, flip angle=10°, in-plane resolution=2.4mm×2.4mm, GRAPPA ~1.65, temporal resolution=162ms, and receiver bandwidth=990Hz/pix. For validation purposes, myocardial T_1_ were compared to reference T_1_ measurements using multi-point SR with TurboFLASH readout (~20s-breath-hold): 1 PDw-image, 12 T_1_w-images with TD 100to600ms every 100ms, then 800to1800ms every 200ms. A nonlinear Levenberg-Marquardt algorithm was used to fit the normalized multi-point SR data. The proposed T_1_-mapping method was also evaluated in a patient with arrhythmia, before and 20min after administrating 0.15mmol/kg Gd-DTPA.

## Results

**Figure 1:**
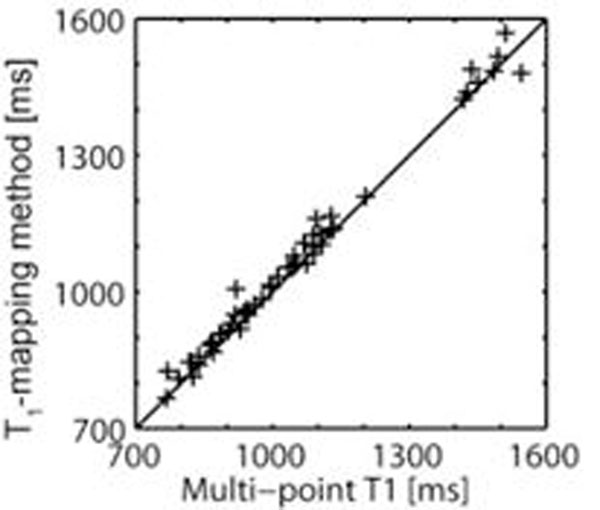
T_1_ measured with the T_1_-mapping method vs. multi-point SR T_1_ measurements in the LV myocardium.

**Figure 2:**
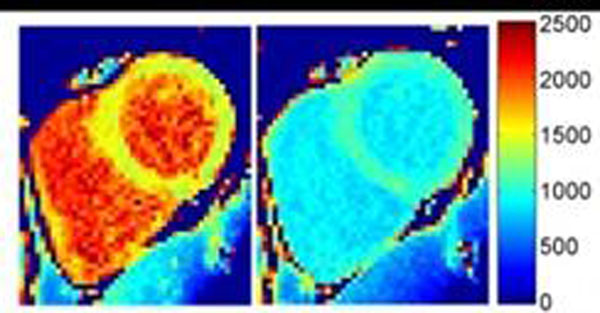
T_1_-maps obtained in a volunteer before and 10 min after 0.05mmol/kg bolus injection.

**Figure 3:**
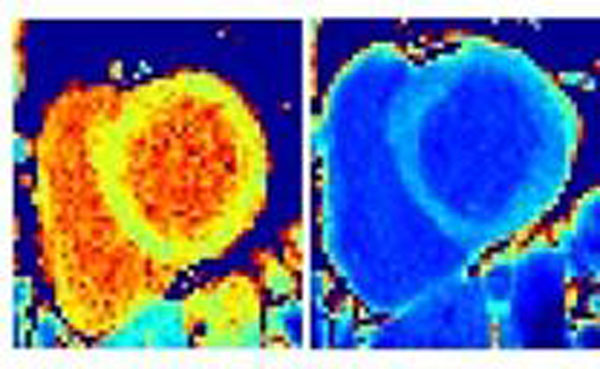
T_1_-maps obtained in a patient with arrhythmias before and 10 min after 0.15mmol/kg slow injection.

Myocardial T_1_ measured using the proposed rapid method were linearly correlated with T_1_ measured using the multi-point T_1_ method (Fig.1, slope=0.99, bias=29ms, r=0.99, P<10^-5^). Pre- and post-contrast T_1_-maps obtained in a 52y.o.-volunteer and a 44y.o.-patient with arrhythmia are shown in Fig.2-3, respectively (same T_1_-scale).

## Conclusion

The proposed T_1_-mapping method is a fast pixel-wise T_1_-mapping technique with insensitivity to cardiac motion and arrhythmia. Future work includes evaluation in patients with acute and chronic infarction.
